# Effects of different gonadotropin preparations in GnRH antagonist protocol for patients with polycystic ovary syndrome during IVF/ICSI: a retrospective cohort study

**DOI:** 10.3389/fendo.2024.1309993

**Published:** 2024-02-12

**Authors:** Zhengyan Hu, Rujun Zeng, Rui Gao, Mingli Chen, Xiumei Liu, Qiong Zhang, Lang Qin, Xun Zeng

**Affiliations:** ^1^ The Reproductive Medical Center, Department of Obstetrics and Gynecology, West China Second University Hospital, Sichuan University, Chengdu, Sichuan, China; ^2^ Key Laboratory of Birth Defects and Related Diseases of Women and Children (Sichuan University), Ministry of Education, Chengdu, Sichuan, China; ^3^ West China School of Medicine, Sichuan University, Chengdu, Sichuan, China; ^4^ Department of Obstetrics and Gynecology, Ziyang Maternal and Child Health Care Hospital, Ziyang, Sichuan, China; ^5^ West China Second University Hospital, Sichuan University, Ziyang Women’s and Children’s Hospital, Ziyang, Sichuan, China

**Keywords:** polycystic ovary syndrome, GnRH antagonist protocol, IVF, ICSI, gonadotropin

## Abstract

**Purpose:**

To compare the effects of recombinant FSH alfa (rFSH-alfa), rFSH-beta, highly purified human menopausal gonadotropin (HP-hMG) and urinary FSH (uFSH) in women with polycystic ovarian syndrome who have undertaken the GnRH antagonist protocol during IVF/ICSI treatment.

**Method:**

A single-center retrospective cohort study including women with PCOS who received the GnRH antagonist protocol from January 2019 to July 2022 was conducted. Patients were divided into rFSH-alfa group, HP-hMG group, uFSH group, and rFSH-beta group, and the number of oocytes retrieved, clinical pregnancy rate of the fresh cycle (primary outcomes), embryo quality, and severe OHSS rate (secondary outcomes) were compared.

**Results:**

No statistical differences were found among the four groups in fresh cycle clinical pregnancy rate (p=0.426), nor in the subgroup analyses. The HP-hMG group had a smaller number of oocytes retrieved and a higher high-quality D3 embryo rate than the three FSH groups (p<0.05). No statistical differences were found among the four groups in the severe OHSS rate (p=0.083).

**Conclusion:**

For women with PCOS undergoing the GnRH antagonist protocol, the clinical pregnancy rates of fresh IVF/ICSI-ET cycle are similar for all four types of Gn. With a lower risk of OHSS and a similar number of high-quality and available embryos, HP-hMG may have an advantage in the PCOS population.

## 1 Introduction

Polycystic ovary syndrome (PCOS) is an endocrine disorder that affects a great number of females of reproductive age and is the most common cause of anovulatory infertility (1). For treatment, *in-vitro* fertilization/intra-cytoplasmic sperm injection (IVF/ICSI) is usually considered a third-line medical therapy when other ovulation induction therapies have failed (2). However, due to the increased antral follicular count (AFC) and anti-Müllerian hormone (AMH) in PCOS patients, there is an increased sensitivity and response to controlled ovarian stimulation (COS) and therefore a higher risk of ovarian hyperstimulation syndrome (OHSS) (3, 4).

One of the important ways to improve these problems is to explore the best protocol for COS. Previous studies (5, 6) have shown that the gonadotrophin-releasing hormone (GnRH) antagonist protocol is beneficial to patients with PCOS due to its lower risk of OHSS and is recommended for this population according to the ESHRE guideline (7). As a common type of ovulation stimulant, gonadotropins (Gn) play an important role in COS. Recombinant follicle-stimulating hormone (FSH) alfa (rFSH-alfa), recombinant FSH beta (rFSH-beta), highly purified urinary FSH (uFSH), and highly purified human menopausal gonadotropin (HP-hMG) are four common types of Gn preparation that have been used in COS. Previous studies (8–13) have compared these preparations among the general population but haven’t reached an agreement. Some studies (9–11) suggest that the efficacy is comparable or without clinical significance in live birth rate, clinical pregnancy rate, and number of retrieved oocytes, while others find out that the rFSH-alfa (12, 13) or the HP-hMG (8) may have a better efficacy than the others. However, few articles have focused on the specific population that suffered from PCOS and undertook the GnRH antagonist protocol for COS.

This study was designed to compare the effects of the four Gn preparations, including rFSH-alfa, rFSH-beta, uFSH, and HP-hMG, in women with PCOS who have undertaken the GnRH antagonist protocol. It aimed to provide evidence for the selection and optimization of COS protocols for patients with PCOS.

## 2 Materials and methods

### 2.1 Study design

A single-center retrospective cohort study was conducted at West China Second University Hospital, Sichuan University. Women with PCOS who received the GnRH-antagonist protocol for their first IVF/ICSI cycle from January 2019 to July 2022 were included. The study was approved by the Ethics Committee of West China Second University Hospital.

PCOS was diagnosed according to the Rotterdam criteria (14). Infertility was defined as the failure to be pregnant after at least 12 months of regular unprotected sexual intercourse (15). Demographic characteristics (including age, weight, and body mass index (BMI)), clinical characteristics (including type of infertility, duration of infertility, baseline sex hormones, AMH, and AFC), and treatment information (including ovarian stimulation information and IVF/ICSI cycle information) were collected from the electronic medical record management system.

Exclusion criteria included: 1) with infertility factors other than anovulation; 2) with other endocrine diseases (such as thyroid diseases and diabetes mellitus) or immune diseases (such as systemic lupus erythematosus and antiphospholipid syndrome); 3) with a history of recurrent pregnancy loss; 4) with chromosomal abnormalities; 5) cycles with preimplantation genetic testing; 6) without complete clinical information.

### 2.2 Treatment protocol

All the patients received the GnRH antagonist protocol. COS was started on day 2 of the menstrual cycle with rFSH-alfa (GONAL-F; Merck Serono, Italy), HP-hMG (Menopur; Ferring, Germany), uFSH (Lizhu Pharmaceutical Trading Co., China), or rFSH-beta (Puregon; Organon, The Netherlands). The starting dose was 75-300 IU according to the type of Gn, age, BMI, and AMH, and the daily dose during COS remained unchanged unless the serum estradiol (E2) did not increase after 7 days of COS. The pituitary gonadotrophin suppression was started with a GnRH antagonist (Injection Cetrotide acetate, Aeterna Zentaris, Canada) at a dose of 0.25mg/d on the day 6 of COS (fixed protocol), or the day the dominant follicle reached 14mm diameter or serum E2 reached 300pg/ml (flexible protocol). As soon as two follicles ≥ 18mm or three follicles ≥ 17mm diameter were detected, patients received their last GnRH antagonist injection, and final follicular maturation (ovulation trigger) was performed by human chorionic gonadotropin (hCG; Lizhu Pharmaceutical Trading Co., China) at a dose of 8000-10000 IU according to the patient’s weight. For patients with a high risk of OHSS, 4000-5000 IU of hCG was used. For patients with more than 19 follicles ≥11mm diameter detected on the trigger day, 0.2 mg GnRH agonist (Decapeptyl, Ferring, Germany) was used. Endometrium thickness was measured by transvaginal ultrasound on the trigger day before the injection of hCG for a rough assessment of endometrial receptivity.

Oocytes were retrieved by transvaginal ultrasound-guided aspiration 36-38h after the trigger, and oocyte maturity was assessed. IVF and/or ICSI were performed depending on the medical history and male factors. Fertilization was assessed on day 1 after the oocyte retrieval. Embryo quality was evaluated daily after the fertilization assessment, and high-quality embryos and available embryos were identified on day 3 and day 5 after oocyte retrieval. The assessment of embryo quality was done independently by at least two embryologists and was summarized and negotiated until a consensus was reached. Ultrasound-guided fresh embryo transfer (ET) was conducted on day 3 or day 5 following the embryo quality assessment, and the remaining available embryos were frozen. For patients with at least one high-quality embryo, single embryo transfer was performed on the best embryo. For patients without high-quality embryos, single embryo transfer or double embryo transfer was performed on the best one or two embryos. All operations were performed in accordance with the standard guidelines of the Chinese Medical Association by experienced embryologists. All patients received luteal phase support with intramuscular progesterone (60 mg per day) or vaginal progesterone gel (90 mg per day) combined with oral dydrogesterone (20 mg per day).

Pregnancy was assessed by measurement of serum β-hCG concentrations 2 weeks after ET and confirmed by transvaginal ultrasound 4 weeks after ET.

### 2.3 Data collection and outcomes

Baseline information included age, BMI, infertility duration, infertility type, AMH, baseline serum E2, progesterone (P), luteinizing hormone (LH), FSH, testosterone (T), and AFC. Baseline sex hormones, AMH, and transvaginal ultrasound for AFC were examined on day 2 of the menstrual cycle before the start of COS. All measurements of hormones were performed in the same laboratory using competitive chemiluminescent immunoassay (CLIA, Siemens ADVIA CENTAUR). The normal ranges of sex hormones in the follicle phase are shown in **Supplementary Table S1**.

The primary outcomes were the number of oocytes retrieved and the clinical pregnancy rate (per fresh ET cycle). The secondary outcomes included high-quality and available D3 embryo rate (per normal fertilized oocyte) and count, high-quality and available blastocyst rate (per formed blastocyst), and severe OHSS rate (per ovulation induction cycle). Besides, total Gn dose, metaphase II oocytes (MII) count and rate, normal fertilized rate, fresh ET cancellation rate, duration of Gn use, and trigger day information including sex hormones, number of follicles ≥14mm, and single endometrium thickness (half of endometrium thickness) were also collected.

Clinical pregnancy was defined as the presence of a gestational sac under ultrasound 4 weeks after ET. D3 embryos with ≥6 cells and ≤20% fragmentation were regarded as high-quality embryos, and those with ≥4 cells and ≤35% fragmentation were regarded as available. The quality of blastocysts (day 5) was assessed based on the Gardner and Schoolcraft scoring system. High-quality blastocysts included grades AA, AB, BA, and BB blastocysts, while available blastocysts included grades BC and CB and high-quality blastocysts.

### 2.4 Statistical analysis

Patients were divided into 4 groups: rFSH-alfa group, HP-hMG group, uFSH group, and rFSH-beta group. A Kolmogorov-Smirnov test was used to estimate the normality of distribution for continuous variables. Normally distributed variables were presented as mean ± standard deviation (SD) and analyzed by one-way ANOVA, using Dunnett t-test as appropriate. Non-normally distributed variables were presented as median (25^th^-75^th^ percentiles) and analyzed by Kruskal-Wallis one-way ANOVA, using Bonferroni method as appropriate. Categorical variables were presented as number of cases (percentage) and analyzed by chi-square or Fisher’s exact test as appropriate. P-value of less than 0.05 was regarded as statistically significant. Subgroups were divided based on the median of the interested parameters. All analyses were performed using the SPSS version 26.0 (SPSS Inc., Chicago, IL, UPL).

## 3 Results

### 3.1 Baseline characteristics

A total of 771 patients were included in this retrospective study and were divided into rFSH-alfa group (n=375), HP-hMG group (n=105), uFSH group (n=173), and rFSH-beta group (n=118) according to the type of Gn they used.

Baseline characteristics are shown in **Table 1**. There were no statistical differences among the four groups in age (p=0.301), duration of infertility (p=0.574), type of infertility (p=0.397), baseline FSH (p=0.085) and baseline E2 (p=0.524). The BMI of uFSH group was higher than others (p<0.05) and HP-hMG group was higher than rFSH-alfa group (p<0.05). The AMH and baseline LH of the uFSH group were lower than the rFSH-alfa group (p<0.05). The baseline P of the rFSH-beta group was lower than the rFSH-alfa group (p<0.05). The AFC of the HP-hMG and uFSH groups was lower than the rFSH-alfa group (p<0.05), and the uFSH group was lower than the rFSH-beta group (p<0.05).

**Table 1:** Baseline characteristics.

		rFSH-alfa (n=375)	HP-hMG (n=105)	uFSH (n=173)	rFSH-beta (n=118)	P-value
Age (y)		29.0 (27.0-32.0)	29.0 (26.0-32.0)	29.0 (26.0-32.0)	30.0 (27.0-31.0)	0.301
BMI (kg/m^2^)		22.26 ± 2.92	23.35 ± 3.22^*^	24.44 ± 3.21^*^†^^	22.41 ± 3.22^‡^	<0.001
Duration of infertility (y)		3.0 (2.0-5.0)	3.0 (2.0-5.0)	3.0 (2.0-5.0)	2.0 (2.0-4.0)	0.574
Type of infertility [n (%)]						0.397
Primary infertility		258 (68.8)	80 (76.2)	121 (69.9)	78 (66.1)	
Secondary infertility		117 (31.2)	25 (23.8)	52 (30.1)	40 (33.9)	
AMH (ng/mL)		10.12 (6.91-14.54)	8.44 (6.12-12.18)	6.83 (4.38-12.43) ^*^	10.74 (6.54-16.22) ^‡^	<0.001
Baseline sex hormone						
FSH (IU/L)		6.30 (5.30-7.70)	6.60 (5.70-7.70)	6.30 (5.40-7.50)	6.30 (5.60-7.98)	0.085
LH (IU/L)		8.60 (5.60-13.55)	7.50 (5.80-9.95)	6.70 (4.30-10.10) ^*^	8.15 (5.42-11.22)	0.020
E2 (pg/mL)		40.66 (31.48-53.75)	45.55 (34.38-55.80)	39.20 (28.90-52.30)	43.55 (34.25-52.15)	0.524
P (ng/mL)		0.52 (0.38-0.69)	0.42 (0.33-0.61)	0.44 (0.32-0.60)	0.44 (0.29-0.59) ^*^	0.004
T (ng/ml)		0.39 (0.30-0.49)	0.47 (0.37-0.57) ^*^	0.41 (0.30-0.56)	0.37 (0.26-0.51) ^†^	<0.001
LH/FSH		1.42 (0.93-2.13)	1.15 (0.86-1.60)	1.17 (0.66-1.78) ^*^	1.26 (0.81-1.94)	0.010
AFC		21.0 (18.0-25.0)	20.0 (17.0-23.0) ^*^	20.0 (15.0-23.0) ^*^	21.0 (17.2-25.0) ^‡^	<0.001

Data are presented as mean ± SD, median (25^th^-75^th^ percentiles) or number (percentage).

*p<0.05 compared to rFSHα group; †p<0.05 compared to HP-hMG group; ‡p<0.05 compared to uFSH group.

BMI, Body Mass Index; AMH, anti-mullerian hormone; LH, luteinizing hormone; E2, estradiol; P, progesterone; T, testosterone; AFC, antral follicle count.

### 3.2 Outcomes of ovarian stimulation

The ovarian stimulation characteristics are shown in **Table 2**. There were no statistical differences in the type of GnRH antagonist protocol (p=0.379). The starting Gn dose of the uFSH group was the highest, while the two rFSH groups were the lowest (p<0.001). The total Gn doses of the HP-hMG and uFSH groups were higher than the two rFSH groups (p<0.05). Statistically, the duration of Gn use in the HP-hMG and uFSH groups was different from the two rFSH groups (p<0.05). As for the indicators on the trigger day, there were no statistical differences in endometrium thickness (p=0.501). The uFSH group had the smallest number of follicles≥14mm (p<0.05). The rFSH groups had the highest trigger day E2, while the HP-hMG group had the lowest (p<0.05). The HP-hMG group had lower trigger day LH than the rFSH-alfa group and uFSH group (p<0.05). The uFSH group had a lower trigger day P than the two rFSH groups (p<0.05). The HP-hMG group had smaller numbers of oocytes retrieved and MII oocytes than the three FSH groups (p<0.05). The rFSH-alfa group had a higher MII oocyte rate than the rFSH-beta group (p<0.05). There were no statistical differences among the four groups in the severe OHSS rate (p=0.083).

**Table 2:** Outcomes of ovarian stimulation.

		rFSH-alfa (n=375)	HP-hMG (n=105)	uFSH (n=173)	rFSH-beta (n=118)	P-value
GnRH antagonist protocol						0.379
Fixed		278 (74.1)	85 (81.0)	124 (71.7)	87 (73.7)	
Flexible		97 (25.9)	20 (19.0)	49 (28.3)	31 (26.3)	
Starting dose of Gn (IU)		150.00 (125.00-200.00)	200.00 (156.25-225.00) ^*^	225.00 (225.00-300.00) ^*†^	175.00 (150.00-200.00) ^†‡^	<0.001
Duration of Gn use (d)		10.0 (9.0-11.0)	10.0 (9.0-11.0) ^*^	10.0 (9.0-11.0) ^*^	9.0 (9.0-10.0) ^†‡^	<0.001
Total Gn dose (IU)		1500.00 (1275.00-1856.25)	2137.50 (1750.00-2550.00) ^*^	2250.00 (1875.00-2775.00) ^*^	1500.00 (1275.00-1818.75) ^†‡^	<0.001
Trigger day						
No. of follicles ≥14mm		11.5 (9.0-14.0)	10.0 (8.0-13.0)	9.0 (7.0-11.0) ^*†^	11.0 (8.0-13.8) ^‡^	<0.001
E2 (pg/mL)		5175.50 (3197.30-7630.10)	3176.30 (2396.35-3988.10) ^*^	3438.00 (2277.60-5200.30) ^*†^	4775.05 (3052.38-6981.08) ^†‡^	<0.001
P (ng/mL)		1.06 (0.77-1.39)	1.04 (0.79-1.31)	0.86 (0.66-1.08) ^*^	1.03 (0.68-1.41) ^‡^	<0.001
LH (IU/L)		2.20 (1.20-4.10)	1.65 (1.00-2.75) ^*^	2.40 (1.60-3.80) ^†^	2.05 (1.10-3.58)	0.004
Single Em thickness (mm)		5.20 (4.50-6.00)	5.20 (4.50-5.88)	5.10 (4.50-6.00)	5.00 (4.50-5.88)	0.523
No. of oocytes retrieved		17.0 (12.0-21.2)	11.5 (9.0-15.0) ^*^	12.0 (9.0-18.0) ^*†^	16.0 (10.0-21.8) ^†^	<0.001
No. of MII oocytes		14.0 (10.0-18.0)	10.0 (7.2-12.0) ^*^	12.0 (7.0-16.0) ^*†^	12.5 (8.0-18.0) ^†^	<0.001
MII oocyte rate [n(%)]		5665/6652 (85.2)	1044/1243 (84.0)	2144/2582 (83.0)	1611/1948 (82.7) ^*^	0.014
Severe OHSS rate [n(%)]		12 (3.2)	0 (0.0)	1 (0.6)	3 (2.5)	0.083

Data are presented as median (25^th^-75^th^ percentiles) or number (percentage).

*p<0.05 compared to rFSHα group; †p<0.05 compared to HP-hMG group; ‡p<0.05 compared to uFSH group.

GnRH, gonadotropin releasing hormone; LH, luteinizing hormone; E2, estradiol; P, progesterone; Em, endometrium; MII, metaphase II oocytes; OHSS, ovarian hyperstimulation syndrome.

### 3.3 Outcomes of IVF/ICSI treatment

The IVF/ICSI treatment outcomes were shown in **Table 3**. The normal fertilized rate of the uFSH group was lower than that of the rFSH-alfa group and the HP-hMG group (p<0.05). The uFSH group had a smaller number of high-quality D3 embryos than the two rFSH groups (p<0.05), and the rFSH-alfa group had a larger number of available D3 embryos than the HP-hMG group and uFSH group (p<0.05). The HP-hMG group had a higher high-quality D3 embryo rate than the rFSH-alfa group and the uFSH group (p<0.05), and the highest available D3 embryo rate, high-quality blastocysts rate, and available blastocyst rate (p<0.05). The rFSH-beta group had a lower high-quality blastocyst rate than the rFSH-alfa group (p<0.05), but a higher available blastocyst rate than the rFSH-alfa group and uFSH group (p<0.05). The two rFSH groups had a higher ET cancellation rate than the HP-hMG group (p<0.05), and the rFSH-alfa group had a higher ET cancellation rate than the uFSH group (p<0.05). There were no statistical differences among the four groups in clinical pregnancy rate (p=0.426).

**Table 3:** Outcomes of ART treatment.

		rFSH-alfa (n=375)	HP-hMG (n=105)	uFSH (n=173)	rFSH-beta (n=118)	P-value
ART method [n (%)]						0.012
IVF		310 (82.7)	92 (87.6)	148 (85.5)	102 (86.4)	
ICSI		17 (4.5)	11 (10.5)	10 (5.8)	5 (4.2)	
IVF+ICSI		48 (12.8)	2 (1.9) ^*^	15 (8.7)	11 (9.3)	
Normal fertilized rate [n(%)]		4120/6652 (61.9)	787/1243 (63.3)	1464/2582 (56.7) ^*†^	1153/1948 (59.1)	<0.001
No. of high-quality D3 embryos		4.0 (2.0-7.2)	4.0 (2.0-6.0)	3.0 (1.0-5.0) ^*^	4.5 (2.0-7.0) ^‡^	<0.001
High-quality D3 embryos rate [n(%)]		1971/4120 (47.8)	426/787 (54.1) ^*^	669/1464 (45.7) ^†^	582/1153 (50.5)	0.001
No. of available D3 embryos		9.0 (6.0-13.0)	7.0 (6.0-9.0) ^*^	6.0 (4.0-10.0) ^*^	8.0 (5.0-12.0)	<0.001
Available D3 embryos rate [n(%)]		3820/4120 (92.7)	775/787 (98.5) ^*^	1339/1464 (91.5) ^†^	1079/1153 (93.6) ^†^	<0.001
High-quality blastocysts rate [n(%)]		679/2199 (30.9)	190/447 (42.5) ^*^	195/658 (29.6) ^†^	151/598 (25.3) ^*†^	<0.001
Available blastocysts rate [n(%)]		1509/2199 (68.6)	426/447 (95.3) ^*^	480/658 (72.9) ^†^	505/598 (84.4) ^*†‡^	<0.001
Fresh ET cancellation rate [n(%)]		288 (76.8)	54 (51.4) ^*^	101 (58.4) ^*^	85 (72.0) ^†^	<0.001
Clinical pregnancy rate [n(%)]		50/87 (57.5)	32/51 (62.7)	38/72 (52.8)	15/33 (45.5)	0.426

Data are presented as median (25^th^-75^th^ percentiles) or number (percentage).

*p<0.05 compared to rFSHα group; †p<0.05 compared to HP-hMG group; ‡p<0.05 compared to uFSH group.

ART, assisted reproductive technology; IVF, in vitro fertilization; ICSI, intracytoplasmic sperm injection; D3, day 3.

### 3.4 Outcomes of subgroup analyses

As was shown in **Table 4**, when dividing the subgroups by age, BMI, weight, LH/FSH, AMH, and AFC, there was no significant difference among the four groups in clinical pregnancy rate in each subgroup. As was shown in **Table 5**, when dividing the subgroups by age, AMH, and AFC, the number of retrieved oocytes was always lower in the HP-hMG group than in the rFSH groups (p<0.05).

**Table 4:** Subgroup analyses for clinical pregnancy rate.

Subgroups based on median		P value
Age	≤ 29	0.320
	> 29	0.580
BMI	≤ 22.6	0.275
	> 22.6	0.082
LH/FSH	≤ 1.26	0.865
	> 1.26	0.360
AMH	≤ 9.5	0.870
	> 9.5	0.146
AFC	≤ 20	0.626
	> 20	0.649

**Table 5:** Subgroup analyses for No. of oocytes retrieved.

Subgroups based on median		rFSH-alfa	HP-hMG	uFSH	rFSH-beta	P value
Age	≤ 29	17.0 (12.0-23.0)	11.0 (8.0-14.0) ^*^	14.0 (8.0-20.0) ^*†^	16.5 (10.0-22.0) ^†^	<0.001
	> 29	16.0 (12.0-20.0)	12.0 (9.2-16.0) ^*^	13.0 (10.8-18.0) ^*^	16.0 (10.0-20.0) ^†^	<0.001
AMH	≤ 9.5	14.0 (10.0-18.0)	11.0 (8.0-16.0) ^*^	12.0 (8.5-17.0) ^*^	14.0 (9.0-20.0) ^†‡^	<0.001
	> 9.5	18.0 (14.0-24.0)	12.0 (9.8-14.2) ^*^	17.5 (12.0-24.0) ^†^	17.0 (10.0-22.0) ^†^	<0.001
AFC	≤ 20	15.0 (11.0-20.0)	11.0 (9.0-14.0) ^*^	12.0 (9.0-16.5) ^*^	15.0 (9.0-21.0) ^†^	<0.001
	> 20	18.0 (14.0-24.0)	13.0 (10.0-16.0) ^*^	16.0 (9.8-22.5) ^*†^	16.5 (12.0-20.0) ^†^	<0.001

Data are presented as median (25^th^-75^th^ percentiles) or number (percentage).

*p<0.05 compared to rFSHα group; †p<0.05 compared to HP-hMG group; ‡p<0.05 compared to uFSH group.

## 4 Discussion

This is a single-center retrospective cohort study concerning the effects of four different types of Gn on women with PCOS undergoing the GnRH antagonist protocol. In this study, we mainly used the number of retrieved oocytes and the clinical pregnancy rate to assess the efficacy of COS and the fresh IVF/ICSI-ET cycle. We found that the lowest number of oocytes retrieved was observed in the HP-hMG group and the highest number in the two rFSH groups. The HP-hMG group had the highest high-quality embryo rate, while the rFSH-alfa group had the highest high-quality embryo number. Though there was no significant difference among the four groups in clinical pregnancy rate, it seemed that the HP-hMG group had the highest clinical pregnancy rate numerically.

The four Gn preparations are different in production and composition. The two rFSH preparations are synthesized by the same recombinant DNA technology but differ in the glycosylation and purification procedures. They are considered to be 99% pure FSH, without LH activity (10, 16). The hMG and uFSH are human-derived preparations from the urine of postmenopausal women and contain <5% copurified proteins. The hMG contains FSH and LH activity in a 1:1 ratio, and the uFSH mainly contains FSH, with a little LH activity due to the purification process (10). The LH activity of hMG derives from LH itself and/or hCG, and in this study, Menopur is an HP-hMG preparation whose LH activity mainly derives from hCG content (17). The differences among the four Gn preparations in composition and production lead to differences in biological activity and effect, which may influence the efficacy of COS and IVF/ICSI-ET.

In the PCOS population, the number of retrieved oocytes during COS tends to be excessive (18). In this study, we found that the use of HP-hMG led to a significantly lower number of retrieved oocytes compared to other Gn preparations. The uFSH group also retrieved fewer oocytes than the rFSH groups. The result for HP-hMG is in agreement with previous studies (19) but the result for uFSH is not (9, 10). It may be because previous studies didn’t focus on the PCOS population or the GnRH antagonist protocol (9, 10, 19). Besides, AMH and AFC have been suggested as predictors of the number of oocytes retrieved (20). In this study, the baseline AFC and AMH of the uFSH group were statistically lower than the two rFSH groups, which may affect the result. The baseline AFC of the HP-hMG group was also different from the rFSH-alfa group, but this difference seemed not to be enough to explain the difference in the oocyte retrieved number.

More oocytes retrieved may be related to a higher risk of severe OHSS, as reported in previous studies (21–23). In this study, though without statistical difference, the severe OHSS rate is numerically consistent with the number of oocytes retrieved. Therefore, the use of HP-hMG may lead to a lower risk of severe OHSS than the three FSH groups. According to our subgroup analyses divided by medians of age, AMH, and AFC, this tendency existed in all the subgroups, especially in patients with higher AMH and/or AFC. It has also been indicated that more oocytes retrieved may be correlated with a higher E2 level on the trigger day (24, 25). Our results also show this trend. E2 is mainly produced by mature follicles that are more than 8mm diameter (26), and may be able to reflect the number and size of mature follicles to some extent. The excessive E2 level during the COS is generally considered a risk for OHSS (27), and it may have a concentration-dependent effect on the pregnancy and birth outcomes (such as clinical pregnancy rate, live birth weight, and preeclampsia) in the fresh IVF-ET cycle (25, 28, 29). Therefore, clinicians tend to withhold Gn or cancel the fresh cycle if excessive E2 levels are observed (30). This was shown in our results as the consistency of the number of oocytes retrieved, trigger day E2, fresh cycle cancellation rate, and severe OHSS rate.

Apart from OHSS, the number of oocytes retrieved is also considered a positive predictor of live birth. Previous studies suggested that the fresh live birth rate seemed to be maximized when the retrieved oocytes reached a plateau whose lower limit ranged from 6 to 11 and the upper limit ranged from 15 to 20 (18, 21, 22, 31–33). The cumulative live birth rate, however, was indicated to increase continuously with the number of oocytes retrieved and stabilize after the number of 20 (22, 31). Therefore, according to our results, the HP-hMG group seems to be more beneficial for the fresh cycle, and the rFSH groups seem to be better for the cumulative live birth rate. However, the optimal range derived from these studies has a large variation due to differences in COS protocols, populations, and grouping, so this finding needs further validation. The exploration of the optimal range also needs to take the risk of OHSS into account. Besides, it is worth mentioning that, though with the lowest number of oocytes retrieved, the HP-hMG group has the highest proportion of high-quality and available embryos, and it obtains a statistically similar number of high-quality D3 embryos as the rFSH groups. It may be due to the HCG-driven LH activity of Menopur, which may produce hormone changes beneficial for embryo quality (34). Therefore, for patients with PCOS, HP-hMG might have an advantage. Previous studies concerning the effectiveness of different Gn preparations showed a better live birth rate and cumulative live birth rate in people using rFSH-alfa for COS (12, 13). However, studies focused on the PCOS population and the GnRH antagonist protocol should be conducted to explore this issue. Therefore, further follow-up and more research are needed.

In this study, we did not find a significant difference in clinical pregnancy rates in the fresh cycle between the four preparations, nor in subgroup analyses divided by medians of age, BMI, LH/FSH, AMH, or AFC. This result is consistent with a meta-analysis in 2019 that focused on the PCOS population (35). However, numerically, the clinical pregnancy rate in the HP-hMG group was the highest, while in the rFSH-beta group it was the lowest. The small sample size of the fresh cycle might limit the exploration, and a high-quality randomized clinical trial (RCT) is still needed to validate this trend.

We also found that the total dose of rFSH during the COS process was less than the urinary preparations, which may be because the urinary preparations are more acidic and therefore somewhat less potent than the recombinant preparations (36). Besides, due to the better stability and liquid formulations of the recombinant preparations, pen injection devices have been used for administration, which allows more precise dose adjustment of 25IU or 12.5IU, possibly leading to a smaller dosage (37). In addition, due to the low batch-to-batch variability, rFSH-alfa (GONAL-F) is able to be provided filled-by-mass, while other preparations, including rFSH-beta, are still filled-by-bioassay (38). Therefore, though without statistical difference, the total dose and clinical efficacy of the rFSH-alfa preparation may be more stable than the rFSH-beta preparation (39). Considering that the price per unit of the urinary preparations is usually slightly lower than that of the rFSH preparations in most countries, the economic analysis needs to take into account the specific conditions of different countries and regions.

This study was conducted at West China Second University Hospital, Chengdu, China. On the one hand, this is an authoritative hospital in China, and the embryo laboratory in our center is built in strict accordance with national standards, with regular quality control of equipment, environment, and technical operations to ensure the stability of medical quality and scientific results. On the other hand, the patients in our center come from a wide range of areas, and their baseline characteristics are representative of the Chinese and East Asian populations. It is worth mentioning that, compared to Caucasian patients, a lower BMI has been reported in East Asian patients with PCOS, which our data of 22.6 kg/m^2^ in median is close to (40). Therefore, we believed that more well-designed studies in the future in different regions, taking into account differences in ethnicity, cultural environment, dietary habits, and so on, would help to provide evidence for Gn use in COS in a wider population.

The study does have some limitations. Firstly, it is a single-center retrospective cohort study and may have some bias, especially in the inclusion of patients. Secondly, the differences in sample size between groups may have affected the statistical differences in some indicators. Thirdly, some baseline characteristics were not all statistically identical, especially the difference between the uFSH group and the rFSH groups, which may be confounding factors. Fourthly, only fresh cycles were included in this study, for they are temporally close to the COS process and have a high likelihood of being affected by gonadotropins. Besides, in order to focus on fresh cycle outcomes, the small sample size for clinical pregnancy rate may have limited the results. In the future, frozen cycles may be included to explore the effect of different Gn preparations on the cumulative pregnancy rate and cumulative live birth rate. Fifthly, not all the patients received single embryo transfer. Double embryo transfer was performed for patients without high-quality embryos, which tended to lessen the impact of embryo quality on clinical pregnancy rates. Therefore, well-designed RCTs are still needed for further exploration of pregnancy outcomes in the future.

## 5 Conclusion

In conclusion, for women with PCOS undergoing the GnRH antagonist protocol, use of the four types of Gn leads to a similar clinical pregnancy rate in the fresh IVF/ICSI-ET cycle, but it seems that the use of HP-hMG leads to the highest clinical pregnancy rate numerically. In addition, the use of HP-hMG leads to a lower number of retrieved oocytes than others and therefore seems to have a lower risk of OHSS. Overall, HP-hMG may have an advantage in the PCOS population. The results in this study need to be proven by further follow-up and well-designed RCTs or prospective studies in the future.

## Data availability statement

The raw data supporting the conclusions of this article will be made available by the authors, without undue reservation.

## Ethics statement

This study involving humans was approved by Ethics Committee of West China Second University Hospital and written informed consent was waived. The study was conducted in accordance with the local legislation and institutional requirements.

## Author contributions

ZH: Writing – original draft, Formal Analysis. RZ: Writing – original draft, Data curation. RG: Writing – review & editing. MC: Writing – review & editing, Data curation. XL: Writing – review & editing, Data curation. QZ: Writing – review & editing, Data curation. LQ: Writing – review & editing, Conceptualization. XZ: Writing – review & editing, Supervision.
