# Supplementary material for: Effects of different gonadotropin preparations in GnRH antagonist protocol for patients with polycystic ovary syndrome during IVF/ICSI: a retrospective cohort study
Source: Front Endocrinol (Lausanne). 2024 Feb 12;15:1309993. doi: 10.3389/fendo.2024.1309993 (PMC10895441; doi:10.3389/fendo.2024.1309993)
Supplement: Supplementary file 1 [file Table_1.docx]

Supplementary Material

# Supplementary Tables

| Table S1 Normal ranges of sex hormones in follicle phase | |
| --- | --- |
| Hormone | Normal range |
| E2 (pg/ml) | 19.5-144.2 |
| P (ng/ml) | 0.15-1.40 |
| LH (IU/L) | 1.9-12.5 |
| FSH (IU/L) | 2.5-10.2 |
| T (ng/ml) | 0.08-0.40 |
| E2: estradiol; P: progesterone; LH: luteinizing hormone; FSH: follicle-stimulating hormone; T: testosterone | |
